# Ileocecal CD5-Positive Diffuse Large B-Cell Lymphoma Presenting with Acute Obstructive Symptoms: A Case Report

**DOI:** 10.70352/scrj.cr.26-0023

**Published:** 2026-04-08

**Authors:** Jumpei Kashiwagi, Yutaka Hanaoka, Hana Yoshimitsu, Masato Wakamatsu, Yasuhiro Takahashi, Daisuke Tomita, Yuika Kureyama, Yusuke Maeda, Naoto Okazaki, Kosuke Hiramatsu, Yudai Fukui, Shigeo Toda, Shuichiro Matoba, Masashi Ueno, Hiroya Kuroyanagi

**Affiliations:** Department of Gastroenterological Surgery, Toranomon Hospital, Tokyo, Japan

**Keywords:** small intestinal lymphoma, diffuse large B-cell lymphoma, CD5-positive lymphoma, obstructive symptoms, ileocecal region

## Abstract

**INTRODUCTION:**

Primary gastrointestinal lymphoma is a rare form of extranodal non-Hodgkin lymphoma, accounting for approximately 1%–4% of all gastrointestinal malignancies. Among primary gastrointestinal lymphomas, involvement of the small intestine accounts for approximately 20%–30% of cases. CD5-positive diffuse large B-cell lymphoma (DLBCL) is a rare variant, representing approximately 5%–10% of all DLBCL cases and is associated with advanced age, extranodal involvement, and poorer prognosis. We report a rare case of ileocecal CD5-positive DLBCL presenting with acute obstructive symptoms.

**CASE PRESENTATION:**

An 80-year-old man presented with abdominal pain and fever. Contrast-enhanced CT demonstrated circumferential wall thickening in the ileocecal region with enlarged regional lymph nodes, resulting in marked luminal narrowing and raising suspicion for locally advanced right-sided colon cancer presenting with acute obstructive symptoms. Emergency laparoscopic right hemicolectomy was performed. Intraoperatively, the tumor appeared hard and showed dense adhesion to the duodenum, with suspected duodenal involvement. The gross specimen revealed an 8 × 7 × 4-cm ulcerated mass arising from the terminal ileum. Histopathology confirmed CD5-positive DLBCL (non-germinal center B-cell type), and regional lymph nodes were histologically negative (0/11). The postoperative course was uneventful, and the patient was referred to the hematology department. PET-CT was initially recommended but deferred by the patient; later imaging suggested disseminated disease, and rituximab, cyclophosphamide, doxorubicin, vincristine, and prednisone immunochemotherapy was initiated, although the long-term outcome could not be confirmed.

**CONCLUSIONS:**

Malignant lymphoma should be considered in the differential diagnosis of acute obstructive symptoms, even when imaging findings suggest carcinoma. Prompt surgical intervention can facilitate definitive diagnosis and timely referral for systemic therapy in selected cases.

## Abbreviations

CD
: cluster of differentiation
DLBCL
: diffuse large B-cell lymphoma
EBER
: Epstein–Barr virus–encoded small RNA
EBV
: Epstein–Barr virus
FDG
: fluorodeoxyglucose
FISH
: fluorescence in situ hybridization
MUM1
: multiple myeloma oncogene 1 (IRF4)
R-CHOP
: rituximab, cyclophosphamide, doxorubicin, vincristine, and prednisone
sIL-2R
: soluble interleukin-2 receptor

## INTRODUCTION

Primary gastrointestinal lymphoma is a rare form of extranodal non-Hodgkin lymphoma, accounting for approximately 1%–4% of all gastrointestinal malignancies.^1)^ Among these, the small intestine is involved in approximately 20%–30% of cases.^1,2)^ DLBCL represents the most common histologic subtype of small intestinal lymphoma.^1,3)^ CD5-positive DLBCL is an uncommon variant, comprising approximately 5%–10% of all DLBCL cases.^4,5)^ It is associated with adverse clinical features, including advanced age, extranodal involvement, and inferior prognosis.^4–6)^ We herein describe a rare case of ileocecal CD5-positive DLBCL presenting with acute obstructive symptoms.

## CASE PRESENTATION

An 80-year-old Japanese man presented with abdominal pain and fever. His medical history included bilateral renal stones and a left femoral fracture. On admission, his blood pressure was 90/62 mmHg, pulse rate 110 beats/min, and body temperature 38.6°C. Physical examination revealed lower abdominal tenderness without peritoneal guarding. Laboratory analyses showed a lactate dehydrogenase level of 186 U/L, C-reactive protein level of 1.50 mg/dL, white blood cell count of 10600/μL, and hemoglobin level of 12.7 g/dL. Serum carcinoembryonic antigen and carbohydrate antigen 19-9 levels were within normal limits. He had no apparent B symptoms such as weight loss or night sweats.

**Fig. 1:**
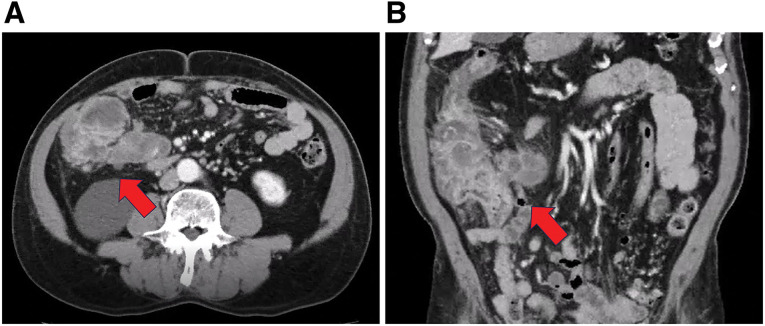
Preoperative contrast-enhanced CT findings. (**A**) Axial and (**B**) coronal contrast-enhanced CT images show a circumferential lesion in the ileocecal region (arrows) with enlarged regional lymph nodes, causing marked stenosis and raising suspicion for locally advanced colon cancer with acute obstructive symptoms.

Contrast-enhanced CT demonstrated circumferential wall thickening of the ileocecal lesion with enlarged regional lymph nodes, resulting in near-occlusive luminal narrowing and raising suspicion for locally advanced right-sided colon cancer (**Fig. 1**). Although marked proximal bowel dilatation or a clearly defined transition point was not evident, the patient had severe abdominal pain with obstipation (minimal stool and flatus), consistent with clinically significant high-grade stenosis and impending complete obstruction.

No ascites or distant metastasis was observed. Colonoscopy was deferred due to concern for worsening obstructive symptoms, and a preoperative histologic diagnosis could not be obtained. Emergency laparoscopic right hemicolectomy was therefore performed.

**Fig. 2:**
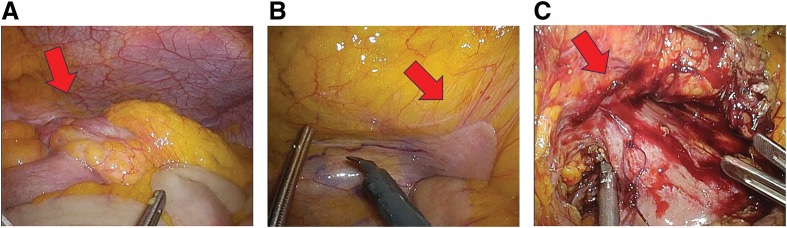
Intraoperative findings. (**A**) Initial laparoscopic view showing a bulging mass-like ileocecal lesion with mild dilatation of the terminal ileum (arrow). (**B**) Before dissection, the duodenum was densely adherent to the mesocolon near the tumor, raising suspicion of invasion (arrow). (**C**) After mobilization of the surrounding tissues, adhesion remained only at the duodenum adjacent to the tumor (arrow).

**Fig. 3:**
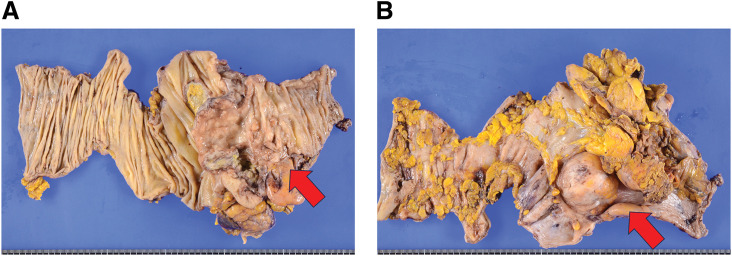
Gross pathological findings. (**A**) Mucosal and (**B**) serosal views of the resected specimen demonstrate an irregular ulcerated tumor arising from the terminal ileum with extension into the cecum. The tumor measured approximately 8 × 7 × 4 cm.

At laparoscopy, the cecum appeared bulged and mass-like, with mild dilatation of the terminal ileum (**Fig. 2A**). The duodenum was densely adherent to the mesocolon adjacent to the tumor before dissection (**Fig. 2B**); after mobilization of the surrounding tissues, adhesion remained only at the duodenum near the tumor (**Fig. 2C**), suggesting duodenal involvement. The tumor appeared hard, and dense adhesion to the duodenum suggested possible invasive disease, which supported the preoperative impression of locally advanced carcinoma rather than lymphoma. Partial resection of the duodenal serosa and reinforcement suturing were performed. Intraoperative upper gastrointestinal endoscopy confirmed the absence of duodenal stenosis. Because cT4b right-sided colon cancer was suspected, an oncologic D3 lymphadenectomy was performed. Gross examination of the resected specimen revealed an irregular, ulcerated mass measuring 8 × 7 × 4 cm arising from the terminal ileum and extending into the cecum (**Fig. 3**). The distance from the ileocecal valve to the proximal resection margin was 10 cm (measured by pathology); because the tumor was centered in the terminal ileum, the proximal margin from the tumor edge was approximately 8.5 cm. The postoperative course was uneventful. Oral intake was resumed on POD 3, and the patient was discharged on POD 11.

**Fig. 4:**
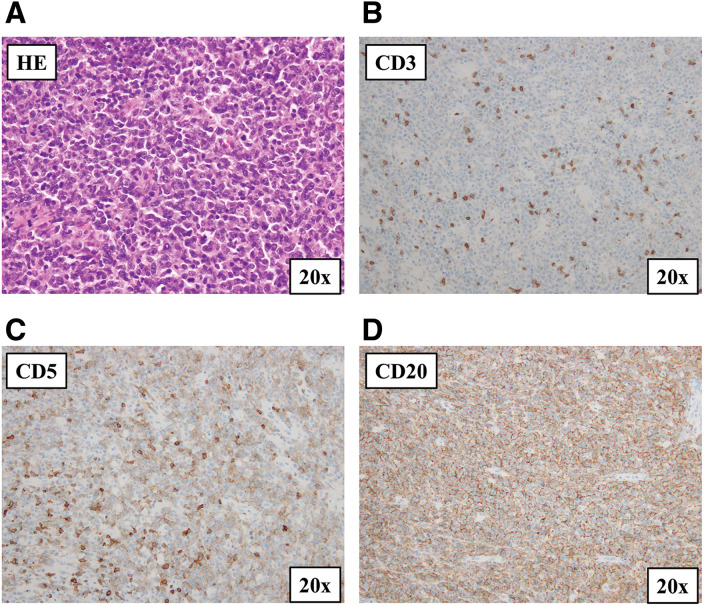
Histopathological and immunohistochemical findings. (**A**) Hematoxylin and eosin staining shows diffuse proliferation of medium-to-large atypical lymphoid cells (×20). Immunohistochemical staining reveals negativity for CD3 (**B**) and positivity for CD5 (**C**) and CD20 (**D**) (×20), consistent with CD5-positive diffuse large B-cell lymphoma. CD, cluster of differentiation; HE, hematoxylin and eosin stain

Final histopathological examination demonstrated diffuse proliferation of medium-to-large atypical lymphoid cells (**Fig. 4A**). Immunohistochemistry showed positivity for CD20, CD5, and MUM1 and negativity for CD3, CD10, cyclin D1, CD23, and SOX11 (**Fig. 4B**–**4D**), consistent with CD5-positive non-germinal center B-cell type DLBCL. The Ki-67 labeling index was 70%–80% in hot spots. BCL2 and BCL6 showed partial positivity, with weak BCL6 expression in <30% of tumor cells. Break-apart FISH analysis revealed no MYC rearrangement (0% split signals) but demonstrated BCL6 (21%) and BCL2 (11%) rearrangements. EBER in situ hybridization showed weak positivity in very few cells. A total of 11 regional lymph nodes were retrieved, and none showed lymphoma involvement (0/11). No duodenal mucosal invasion was observed. Because PET-CT was not obtained in the immediate postoperative period, the Lugano stage at the time of surgery could not be definitively established based on resection pathology and CT alone.^7)^

He was referred to the hematology department, where staging PET-CT was recommended but declined; sIL-2R measured at that time was 469 U/mL. At approximately 2 months postoperatively, he re-presented with sore throat, and PET-CT was performed; sIL-2R at that visit was 1391 U/mL. PET-CT demonstrated FDG uptake in multiple nodal regions (bilateral cervical, left axillary, mesenteric, pelvic, and bilateral inguinal lymph nodes), as well as the pharynx. Marked bone marrow uptake and suspected hepatic involvement were also noted (Deauville score 5, per the nuclear medicine report), suggesting disseminated disease. He was admitted to the hematology department, and R-CHOP was initiated; however, the subsequent long-term outcome could not be confirmed.

## DISCUSSION

Primary small intestinal lymphoma infrequently presents with acute obstructive symptoms; most cases manifest with nonspecific symptoms, and bowel obstruction has been reported only in a minority of case series and case reports.^3,8,9)^ However, severe stenosis and impending obstruction may occur in cases with large tumors or suspected invasion into adjacent organs.

In the present case, preoperative contrast-enhanced CT demonstrated a circumferential ileocecal lesion with enlarged regional lymph nodes, resulting in near-occlusive luminal narrowing at the ileocecal region, findings that closely mimicked advanced colon cancer with deep invasion (**Fig. 1**). CT did not demonstrate marked proximal bowel dilatation or a clearly defined transition point; however, the clinical presentation—severe abdominal pain with fever and minimal passage of stool and flatus—supported the clinical diagnosis of clinically significant high-grade stenosis with impending complete obstruction, rather than radiographically established obstruction. The severity of the abdominal symptoms precluded preoperative endoscopic evaluation. Emergency surgery was therefore undertaken without a definitive preoperative diagnosis. Accordingly, in this manuscript, “impending obstruction” refers to a clinical diagnosis of high-grade stenosis with impending complete obstruction, not radiographically established obstruction.

In acute settings, an ileocecal mass with suspected duodenal involvement is often interpreted as locally advanced colon cancer. In our case, the tumor appeared hard intraoperatively with dense adhesion to the duodenum, which reinforced the impression of carcinoma. Retrospectively, however, prominent lymphadenopathy with vascular displacement rather than overt vascular invasion, and a potentially lobulated and relatively homogeneous mass-like component, could be considered findings that may favor lymphoma. No preoperative laboratory clues such as sIL-2R were available, and there were no clear systemic “B symptoms.”

When clinically feasible, preoperative histologic confirmation is essential. Terminal ileum intubation (ileoscopy) with biopsy during colonoscopy, and balloon-assisted enteroscopy in selected cases, may enable tissue diagnosis and help avoid potentially unnecessary emergent definitive resection. In addition, sIL-2R may provide supportive information when differentiating lymphoma from carcinoma. PET-CT is also important for staging and response assessment in lymphoma, even if it cannot be performed emergently. In the present case, later PET-CT suggested disseminated disease.

In general, once DLBCL is diagnosed and no emergent complication is present, systemic immunochemotherapy is prioritized. However, in intestinal lymphoma, surgical intervention can be appropriate in situations of uncontrolled or impending obstruction, suspected/impending perforation, significant bleeding, or persistent diagnostic uncertainty (e.g., inadequate biopsy). Moreover, tumor regression during chemotherapy may carry a risk of gastrointestinal perforation, necessitating careful shared decision-making in symptomatic cases. In our case, because the lesion was considered resectable, endoscopic biopsy was not feasible, and possible duodenal involvement suggested locally advanced carcinoma, we chose definitive resection; nevertheless, temporary diversion and staged treatment are important alternatives in selected patients.

Gross examination of the resected specimen revealed a large ulcerated tumor centered in the terminal ileum, measuring approximately 8 × 7 × 4 cm (**Fig. 3**). Histopathological examination showed diffuse proliferation of medium-to-large atypical lymphoid cells (**Fig. 4A**). Immunohistochemical analysis demonstrated positivity for CD20, CD5, and MUM1 and negativity for CD3, CD10, and cyclin D1, supporting the diagnosis of CD5-positive non-germinal center B-cell type DLBCL (**Fig. 4B**–**4D**). The high proliferative activity (Ki-67 70%–80% in hot spots) was consistent with the aggressive clinical presentation. Although BCL2 and BCL6 rearrangements were detected by FISH, MYC rearrangement was not observed, and therefore the findings did not suggest a MYC-rearranged high-grade B-cell lymphoma. The very limited EBER positivity was not considered supportive of EBV-positive DLBCL.^10)^ No direct infiltration of atypical lymphoid cells into the duodenal wall was identified, despite intraoperative suspicion of invasion. Given the CD5 expression, mantle cell lymphoma was considered in the differential diagnosis; however, the tumor cells were negative for cyclin D1 and SOX11, supporting the diagnosis of de novo CD5-positive DLBCL.

CD5-positive DLBCL is an uncommon subtype, accounting for approximately 5%–10% of all DLBCL cases, and is associated with a poorer prognosis compared with CD5-negative DLBCL.^4–6)^ Reported adverse prognostic factors include advanced age, large tumor size, extranodal involvement, and non-germinal center B-cell phenotype.^4–6)^ Although the present patient showed a normal serum lactate dehydrogenase level at presentation, several adverse prognostic factors were present, including advanced age, large tumor size, non-germinal center B-cell phenotype, and CD5 expression.

Surgical resection played a crucial role in this case by relieving severe stenosis/impending obstruction and enabling definitive histopathological diagnosis, thereby enabling timely referral for systemic chemotherapy.^11,12)^ Given the aggressive clinical behavior of CD5-positive DLBCL, this case highlights the importance of early recognition and multidisciplinary management involving both surgical and hematological expertise. This report has important limitations. PET-CT was not obtained in the immediate postoperative period; therefore, the clinical stage at presentation could not be definitively established. Later, PET-CT suggested disseminated disease, and R-CHOP was initiated; however, long-term outcome could not be confirmed.

## CONCLUSIONS

We report a rare case of ileocecal CD5-positive DLBCL presenting with acute obstructive symptoms. Malignant lymphoma should be considered in the differential diagnosis of acute obstructive symptoms, even when imaging findings suggest carcinoma. Prompt surgical intervention may be essential for diagnosis and initiation of appropriate systemic therapy when impending obstruction, perforation risk, bleeding, or diagnostic uncertainty is present.

## DECLARATIONS

### Availability of data and materials

No datasets were generated or analyzed during the current study.
